# Biomarkers of Inflammation in Obesity-Psoriatic Patients

**DOI:** 10.1155/2019/7353420

**Published:** 2019-05-28

**Authors:** Carmen Rodríguez-Cerdeira, Mónica Cordeiro-Rodríguez, Miguel Carnero-Gregorio, Adriana López-Barcenas, Erick Martínez-Herrera, Gabriella Fabbrocini, Ardiana Sinani, Roberto Arenas-Guzmán, José Luís González-Cespón

**Affiliations:** ^1^Efficiency, Quality and Costs in Health Services Research Group (EFISALUD), Health Research Institute, SERGAS-UVIGO, Vigo, Spain; ^2^Dermatology Department, Hospital do Meixoeiro and University of Vigo, Vigo, Spain; ^3^European Women Dermatological and Venereological Society (EWDVS), Vigo, Spain; ^4^Department of Molecular Diagnosis (Array & NGS Division), Institute of Cellular and Molecular Studies (ICM), Lugo, Spain; ^5^Psychodermatology Task Force of the Ibero Latin American College of Dermatology, Argentina; ^6^Mycoloy Service, Hospital Manuel Gea González, Mexico City, Mexico; ^7^Research Unit, High Specialty Regional Hospital of Ixtapaluca, Ixtapaluca, Mexico; ^8^Dermatology Service, University of Napoli Federico II, Naples, Italy; ^9^Dermatology Service, Military Medical Unit, University Trauma Hospital, Tirana, Albania

## Abstract

Psoriasis is a common chronic inflammatory multisystemic disease with a complex pathogenesis consisting of genetic, immunological, and environmental components. It is associated with a number of comorbidities, including diabetes, metabolic syndrome, obesity, and myocardial infarction. In addition, the severity of psoriasis seems to be related to the severity of obesity. Patients with higher levels of obesity show poorer response to systemic treatments of psoriasis. Several studies have demonstrated that white adipose tissue is a crucial site of the formation of proinflammatory adipokines such as leptin, adiponectin, and resistin and classical cytokines such as interleukin- (IL-) 6 and tumour necrosis factor-*α*. In psoriasis, due to the proliferation of Th1, Th17, and Th22 cells, IL-22, among others, is produced in addition to the abovementioned cytokines. With respect to leptin and resistin, both of these adipokines are present in high levels in obese persons with psoriasis. Further, the plasma levels of leptin and resistin are related to the severity of psoriasis. These results strongly suggest that obesity, through proinflammatory pathways, is a predisposing factor to the development of psoriasis and that obesity aggravates existing psoriasis. Different inflammatory biomarkers link psoriasis and obesity. In this paper, the most important ones are described.

## 1. Introduction

Currently, obesity is considered a chronic, multifactorial disease and a result of interactions between genetic load and the environment that affect a large percentage of the population across all ages, sexes, and social conditions. It is defined as an abnormal or excessive accumulation of adipose tissue that can be harmful to health, and it affects a greater proportion of women than men [1].

It is characterized by an elevation of plasma levels of proinflammatory cytokines, including tumour necrosis factor-alpha (TNF-*α*), interleukin-6 (IL-6), and acute-phase proteins such as C-reactive protein (CRP). This condition associated with obesity is explained by the inflammatory activity of adipocytes. Adipose tissue, classically considered as an energy reservoir, is able to communicate with the rest of the body by secreting adipokines, which are molecules with proinflammatory, thrombotic, and vasoactive activity [2]. Adipokines include TNF-*α*, plasminogen activator inhibitor 1, IL-6, and leptin [3].

A decrease in adiponectin, a cytokine with anti-inflammatory activity, has also been observed. In response to these signals, macrophages are attracted to adipose tissue. Once infiltrated trapped between adipocytes, mature macrophages stimulate cytokine secretion, leading to local primary inflammation. Subsequently, cytokines trigger the production of inflammatory proteins in the liver and thus lead to the low-grade systemic inflammation observed in obesity [4]. In addition, cytokines increase lipolysis, which is the constant release of free fatty acids by adipose tissue into peripheral circulation.

Thus, glycoprotein 130 cytokines have presented different effects on adipogenesis, lipolysis, insulin sensitivity, and food intake. In this review, we have summarized the current knowledge about gp130 cytokines, including IL-6, LIF, CNTF, CT-1, and OSM, in adipocyte biology and metabolic activities in conditions such as obesity and type 2 diabetes [5].

Free fatty acids have been considered an important link between chronic inflammation and adipose tissue activity, as they are capable of increasing oxidative stress and, therefore, the inflammatory environment and vascular activity. It should be noted that adipose tissue of central predominance is associated with a greater amount of visceral fat, compared with its peripheral distribution. Adipocytes of visceral fat are metabolically more active, releasing more cytokines and fatty acids. As such, we would expect to find a more pronounced inflammatory environment in patients with abdominal obesity [6].

Psoriasis is a chronic autoimmune disease of genetic predisposition and multifactorial triggers that affects the skin, semi-mucosa, mucosa, annexes, and joints [7]. Globally, it affects 2%-5% of the population [8].

The interactive relationship between hyperproliferative keratinocytes (KCs), inflammatory dendritic cells (DCs), neutrophils, mast cells, and T cells leads to the apparition of psoriatic lesions. These lesions, from the clinical point of view, are characterized by sharply demarked, erythematous, and scaly plaques. They are found predominantly in the scalp, knees, elbows, lumbosacral area, and body folds, with symmetrical distribution, and can develop in sites of injury (Koebner phenomenon). At the moment, there are no differential diagnostic criteria for psoriasis, and its diagnosis is based on examination of the morpho- and histological characteristics of the lesions, clinical history of patients, and the psoriasis area and severity index (PASI). It is usually accompanied by other morbidities that can further affect the quality of life and survival of patients [9].

Therefore, epidemiological studies have identified a greater risk of development of metabolic alterations in these patients, among which obesity is highlighted [10, 11].

Obesity and psoriasis are linked by a common pathophysiological mechanism, which is explained by low-grade chronic inflammation [12]. Obesity is not only associated with a higher incidence and severity of psoriasis, but it also affects the response to treatment [13]. The inflammatory state associated with obesity [14, 15] has been proposed as a link between various pathological conditions that usually coexist, a condition known as “metabolic syndrome,” another comorbidity of psoriasis [16, 17].

The effect of cytokines on insulin sensitivity in the liver and muscle has been widely studied [18]. Unlike psoriasis, its association with obesity during the last decades has been demonstrated at a clinical level, without an extensive study of all the molecular mechanisms involved in this association. However, both diseases are inflammatory pathologies, with a common pathophysiological substrate, such as inflammatory pathways and cytokine accumulation [18].

It is known that tumour necrosis factor alpha (TNF-*α*)—a cytokine that is elevated in patients with psoriasis, rheumatic diseases, and obesity—induces insulin resistance through various mechanisms. Exposure of cells to TNF-*α* causes inhibitory phosphorylation by receptor 1 of TNF-*α* (TNF-R1) to the serine residues of substrate 1 of the insulin receptor (IRS-1), favouring development of insulin resistance [19].

Insulin resistance contributes to the pathogenesis of the metabolic syndrome by generating hyperglycaemia and compensatory hyperinsulinaemia. This favours development of obesity, hepatic steatosis, dyslipidemia, atherosclerotic disease, and, eventually, diabetes mellitus type 2 [20, 21].

**Figure 1:**
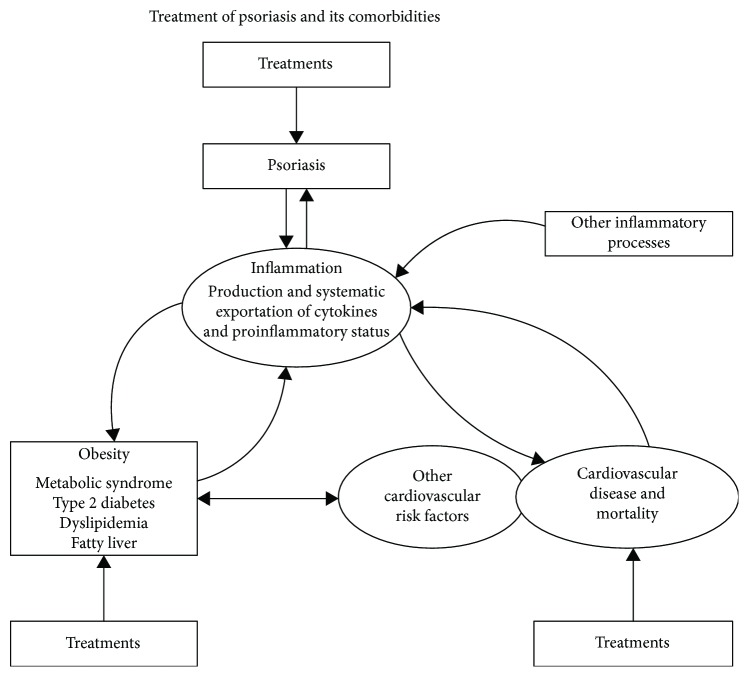
Metabolic pathway of lipoinflammation. The interactive association among psoriasis, obesity, type 2 diabetes, cardiovascular disease, and mortality would be based on the inflammation observed in each of these diseases and transported systemically, especially to other inflammatory processes.

Obesity and psoriasis share common pathogenic mechanisms, including increased proinflammatory cytokines (IL-1, IL-6, TNF-*α*, and adiponectin). Several studies have shown that control of these pathologies favours good evolution of psoriasis and concurrently that treatment with methotrexate and anti-TNF would reduce the risk of these comorbidities [22] (Figure 1).

Therefore, TNF-*α* is probably one of the cytokines responsible for the increased risk of cardiovascular disease experienced by patients with psoriasis. TNF-*α* and IL-1*β* inflammatory cytokines are central mediators of immunity and are involved in cytokines, monoclonal antibodies that target cell surface proteins and receptors. Examples of anti-TNFs are infliximab, etanercept, adalimumab, certolizumab, and golimumab. Examples of monoclonal Abs include ustekinumab, secukinumab, and ixekizumab. Most of them are subcutaneous treatments. They are expensive treatments, and systemic immunosuppression can lead to infections and disease recurrence if there is a discontinuous pattern of treatment. There is also a drug delivery treatment, apart from phototherapy and biological treatment [23], based on two pathways: either to normalize the keratinocyte differentiation or to modulate immune responses.

In psoriasis, the main expressed component of biomarkers is related to hyperproliferation of keratinocytes. This is why the level of certain proteins allows distinguishing between psoriatic and normal skin. Certain biomarkers are still unknown, and other predictions are made based on similarities with other diseases.

In this manuscript, the main diagnostic, prognostic, and treatment response biomarkers were collected in obesity-psoriatic patients.

## 2. Correlation between Obesity—Body Weight and Body Mass Index (BMI)—and Severity of Psoriasis and Response to Treatment

In this review, Alotaibi aimed to study the effects of weight loss on the symptoms of psoriasis in obese patients. Using Ovid, the search since 1990 to December 2017 yielded 14 results [24]. Debbaneh at al. [25] conducted a literature review of observational and clinical literature on the effects of weight loss on the severity of psoriasis. Naldi et al. [26]conducted a randomized controlled trial on 303 patients, which incorporated 20 weeks of dietary and exercise interventions as an adjunct treatment for obese and overweight patients with psoriasis. All of these studies showed significantly improved PASI scores in the group with intervention compared to those in the control group. Al-Mutairi and Nour [27] observed in 2014 that body weight reduction could improve PASI scores in a trial of 262 obese patients on anti-TNF-*α* biologic therapy.

Both psoriasis and obesity are related to an underlying common cause of inflammation. A review of published literature clearly shows that diet and exercise will be considered as adjunct treatments for psoriasis, as they are easily accessible and inexpensive. Weight loss improved the overall health of a patient and was effective in combating oxidative stressors, with secondary positive impacts on the PASI scores. Therefore, the authors recommend that physicians encourage their patients to follow a healthier lifestyle aimed at following an exercise regimen and reducing weight as a method to improve psoriasis symptoms [24].

A meta-analysis was performed with results by Budu-Aggrey et al. [28], using both adults' and children's data separately and in combination. Investigating causal relationships, the analysis included 753,421 individuals from the UK Biobank and Nord-Trøndelag Health Study (HUNT), Norway. A two-sample MR was performed with 356,926 individuals from the published body mass index (BMI) and psoriasis genome-wide association studies (GWASs). For the observational analysis, logistic regression models were used to estimate the observational association between BMI and psoriasis.

Briefly, 56 studies reporting data about the relationship between psoriasis and BMI, obesity, or being overweight were identified. Among them, 35 compared BMI between psoriasis cases and controls, which were considered to be meta-analysed. It was then found a significant difference in BMI between cases and controls of 1.26 kg/m^2^ (95% CI 1.02–1.51) in adults (69,844 psoriasis cases and 617,844 controls) and 1.55 kg/m^2^ (95% CI 1.13–1.98) in children (5–18 years old). In the additional 21 studies, the researchers sought to find an association between BMI and obesity, which reported a positive association.

The Genetic Risk Score for Body Mass Index (BMI GRS) was strongly associated with BMI in the UK Biobank and HUNT [29]. In both UK Biobank and HUNT, a higher BMI was associated with increased risk of psoriasis [30].

Higher BMI increases the risk of psoriases, both diseases, presenting a rising prevalence [31]. Type 2 diabetes and obesity were associated with a significant increased risk of liver fibrosis. The association between psoriasis and obesity should be properly considered when choosing a systemic treatment, because it could exert negative effects on metabolic parameters, including liver enzymes, serum lipids, and renal function. Obesity may increase the risk of liver and renal toxicity from methotrexate and cyclosporine [28].

As reported by Carrascosa et al. [32], obese patients with psoriasis have a higher risk of adverse effects with conventional systemic drugs. Biological drugs in which the dose is not adjusted to weight, such as etanercept and adalimumab, are usually less effective, whereas other biological drugs, such as infliximab and ustekinumab, can be adjusted to weight.

Rui et al. [33] showed that psoriatic patients with MS showed much less reduction of IL-17 and IL-6 before and after 10 sections of NB-UVB treatment, respectively, than patients without MS did (*P* < 0.05). Psoriatic patients with MS had poorer improvement in comparison with those without MS using NB-UVB treatment. MS was an independent factor affecting NB-UVB treatment. In addition, psoriatic patients with MS showed much less reduction of systemic biomarkers (interleukin- (IL) 17, TNF-*α*, and IL-6) than patients without MS did. That is, they may need a longer treatment course to achieve improved skin lesions.

Since 2004, it is possible to treat psoriasis with molecules generated by molecular biology using recombinant DNA technology. Biological treatments in psoriasis are directed against cytokines or surface proteins of lymphocytes blocking specific steps in the pathogenesis of psoriasis [34, 35].

The most commonly active principles used in the treatment of psoriasis are described in Table 1 [33–42].

Baerdazzi et al. [43] have studied 33 patients (27 men and 6 women), 25–45 years old, treated with biological therapies, and “nonresponders” to at least two traditional systemic therapies. The results and follow-up were as follows: mean BMI = 30.59 (±6.94) and average PASI = 25.03 (±12.43), obese patients average PASI = 32.36 (±12.79) and patients with grade III obesity average PASI = 44 (±3.37). Differences were not statistically significant in most cases due to the low number of patients. Overweight and obese patients (BMI > 25) were invited to lose weight. Reevaluation of the PASI after 4 months showed that the reductions were attributable, for the most part, to the treatment. However, if weight reduction was obtained, there was a remarkable improvement in the PASI. At the end of the study, BMI did not change in 22 patients, increased in 4, and decreased in 7. Among the 7 patients who lost weight, they achieved a PASI of 75 or higher (statistically significant difference). Finally, weight loss could help improve psoriasis and make the treatments used more effective [43].

As in Puig et al. [44], in this study, the correlation between PASI and WhtR was analysed in a population of 289 patients with psoriasis for which anthropometric measurements were available. It was seen that PASI in 243 patients with a WhtR greater than or equal to 0.5 had a higher median than in the 48 patients with a WhtR less than 0.5. The correlation between WhtR and BMI was 0.86, while that between WhtR and PASI was 0.14. WhtR is an accessible method, with an equal cutoff point for both sexes and good correlation in obesity, cardiovascular disease, and diabetes mellitus. In this study, we have seen in obese patients with psoriasis a better correlation of WhtR with PASI than BMI. For each unit of increase in BMI, the risk of suffering psoriasis increased by 9% and PASI 7%. One factor to consider is that therapy with TNF-*α* contributed to weight gain in patients with chronic plaque psoriasis. Thus, the clearance of adalimumab or ustekinumab was greater in obese patients, and the PASI75 or PASI90 response rate was higher in patients with low weight. In the case of etanercept, patients with lower weight had better response rates to the drug than those with obesity. However, PASI75 response rates at 10 weeks of treatment with infliximab were independent of BMI. Thus, in those patients with psoriasis and obesity, a treatment with infliximab could be more advisable in comparison with other biologics that are administered in fixed doses.

In addition, the response of obese patients with moderate or severe psoriatic plaques to treatment with low-dose cyclosporine showed improvements after loss of passage through diet or bariatric surgery, although there are studies that show a worsening of the disease after surgery or with rapid drops in weight [44].

Petridis et al. [45], in a multicentre, prospective, observational study, examined the impact of risk factors, such as BMI and waist circumference, on quality of life improvement and clinical response in moderate-to-severe plaque-type psoriasis patients treated with infliximab in routine care settings in Greece.

Fleming et al. [46] reviewed 254 articles, of which they included only 9. The sample size was 134,823 psoriasis patients. They included data on age, sex, body mass index (BMI), obesity proportion, and psoriasis area severity index (PASI) score. They found a statistically significant association between the BMI and PASI.

According to Klingberg et al. [47], psoriasis affects 2-3% of the population in Sweden, and 20-30% of these patients develop psoriatic arthritis (PsA), both of which are strongly associated with obesity and the metabolic syndrome (MetS). Obesity increases the risk of developing both psoriasis and PsA, and this is associated with higher disease activity and a poorer treatment response. The aim of this study was to determine the effects of weight loss treatment with very low energy diet (VLED) on the disease activity in joints, entheses, and skin in patients with PsA and obesity.

Briefly, 41 patients with psoriatic arthritis (CASPAR criterion) completed the study, showing a BMI ≥ 33 kg/m^2^ with weight loss treatment with very low energy diet (VLED) and daily intake of 640 kcal, including recommended doses of vitamins, minerals, and other essentials, following an initial period of 12 or 16 weeks. The association between BMI and disease activity at baseline BMI was positively correlated with several measures of disease activity and function. BMI decreased from median 35.2 kg/m^2^ to 29.7 kg/m^2^, and a significant reduction was seen in a majority of the disease activity measures. The improvement of the skin occurred later than for the other disease activity parameters (3 months). The treatment was generally well tolerated without serious adverse events occurring. Significant improvement in disease activity in joints, entheses, and skin and reduction in CRP, PLT, and parameters assessing function were found at the 6-month follow-up.

Data from Giunta et al. [48] indicated that obesity (BMI ≥ 30 kg/m^2^) was a negative cause for psoriasis treatment with etanercept. Thus, an increase in BMI may be a predictor to suspending use of the anti-TNF-*α* drug. Therefore, there is a consensus that a BMI of 25 kg/m^2^ is for a good response to treatment.

In this study by Hansel et al. [49], 30 patients received complete treatment with complete clearance; the treatment was optimally applied with dose spacing up to 21 or 28 days. Patients were divided into two groups in the body mass index. Group A of the body mass index remained at a level of 60%, and group B of 40% had to return to the standard dose. In addition, the time to achieve PASI-100 with the standard dose was also less for group A.

In another study conducted by Prussick et al. [50] with psoriatic patients and controls treated with adalimumab vs. methotrexate, PASI responses through 16 weeks of treatment were related to the BMI represented in three categories (<25 kg/m^2^, 25 to <30 kg/m^2^, and ≥30 kg/m^2^). In normal weight, overweight, and patients at week 16, the respective PASI-75 response rates were 85.0%, 85.7%, and 61.3% with adalimumab; 43.3%, 29.3%, and 26.1% with methotrexate; and 28.6%, 16.7%, and 0% with placebo. Adalimumab was superior in all cases, although the result was also influenced by BMI.

In this study, Takamura et al. [51] aimed to investigate the effects of infliximab, ustekinumab, and secukinumab on body weight (BW) and body mass index (BMI) in patients with psoriasis. This retrospective study examined changes in BW and BMI between patients treated with these biologics. Patients presented similar values of BMI and BW at the beginning of the study. The number of patients was as follows: infliximab (*n* = 18), ustekinumab (*n* = 30), and secukinumab (*n* = 20). The treatment results appeared better in patients treated with ustekinumab and secukinumab.

There is still not much known in patients with a high BMI and high body weight with ixekizumab; however, the results were similar so that a high rate of BMI seemed to decrease the effectiveness of treatment in patients with moderate-to-severe plaque-type psoriasis [52].

Finally, 48 patients were included in a study by Vujic et al. [53], who received apremilast between 1 April 2015 and 19 January 2017 and were evaluated every 4 weeks. Further, we documented the following: age, weight, height, smoking psoriasis area severity index (PASI) scores, and the onset and duration of adverse events (AE). Three patients (6.3%) reached PASI-90, nine (18.8%) PASI-75, and eight (16.7%) PASI-50. Patient weight was inversely correlated with PASI-50.

## 3. Association between Some Obesity Biomarkers and Psoriasis Severity

Recent studies have revealed that there are some possible biological markers that can be used to detect psoriasis and evaluate the prognosis and response to treatment.

### 3.1. Biomarker for Diagnosis

In this work from Yadav et al. [54], a global view of the biomarker for psoriasis protein is made. The signal transducer and activator of transcription (STAT) has a key role in psoriasis because of its involvement in some biological actions related to immune pathways, such as cell division, growth, and apoptosis. It is believed that the JAK/STAT intracellular signalling pathways control inflammatory reactions in psoriasis and other diseases. STAT is a transcriptional protein family (consisting of STAT1, STAT2, STAT3, STAT4, STAT5A/5B, and STAT6) involved in the expression of main cytokines and nuclear transmission of extracellular signals. Various studies have suggested the importance of STAT1 in psoriasis, as its elevated activity (upregulated by hyperphosphorylation of TAT1) appears in psoriatic skin. STAT3 plays another important role because its activation in T cells and keratinocytes is involved in the pathogenesis of psoriasis. The high expression of STAT2 is significant in psoriatic lesions. Psoriasis has also a characteristic raised level of IFN*γ*-producing Th1 cells and IL-17A-producing Th17 cells.

Following with S100 proteins, there are 18 S100 family proteins. Among them, 13 are responsible for cellular differentiation of the epidermis and located on chromosome 1q21. In psoriasis, S100A7, S100A8, and S100A9 are expressed. S100A7 (Psoriasin) is involved in abnormal keratinocyte differentiation. Its overexpression is noticed in the nucleus and cytoplasm in keratinocytes and secretion from epithelial cells in psoriatic skin. S100A8 and S100A9 are very significant in psoriatic arthritis and rheumatoid arthritis as they are strongly expressed in inflamed tissue fluid. This is the reason why S100A8 and S100A9 are strong protein candidates for therapeutic targeting of psoriasis and PsA. Another important biomarker is Wnt5a, which is a transmission protein that has a key role in carcinogenesis and embryogenesis and regulates tissue regeneration in the intestine and skin. Wnt5a and its receptors fzd3 and fzd5 have a central role in adult skin cell differentiation and are located in hair follicles of normal epidermal skin, signifying their crucial role in skin cell differentiation in adults. In psoriasis, Wnt5a and fzd5 are overexpressed and relocated, which leads to anomalous differentiation of keratinocytes.

p53/TP53 or tumour protein, one key factor of psoriasis, is a phosphoprotein that regulates cell cycle. Its presence in the layers of psoriatic skin suggests its involvement in the disease.

As for enzyme biomarkers, exfoliations of epidermal cells followed by hyperproliferation are a distinct indication in psoriasis. The development, repair, and proliferation of keratinocytes are controlled by several proteins, enzymes, ions, cytokines, and growth factors. Targeting psoriasis with enzymes could be a unique treatment strategy as they offer insight into the disease progression by prognosis, diagnosis, and assessment of responses. Phospholipase C (PLC) has a key role in the formation of the stratum corneum barrier and keratinocyte differentiation.

Although psoriasin (S100A7) and koebnerisin (S100A15) are distinct in tissue distribution, regulation, and function, both have functional roles in innate immunity, epidermal cell maturation, and epithelial tumourigenesis [55].

Ekman et al. [56] suggest that IL-22 links the inflammatory response to differentiation of immature cells and epithelial regeneration by acting directly on keratinocytes to promote cell stemness. Additionally, IL-22 may have a very important role in the triggering of psoriasis. IL-22 is found in the dermal infiltrate of psoriasis plaques, as well as in the blood of patients with psoriasis.

Importantly, banal infections or small lesions persisting over a long period of time may stimulate IL-22 production. According to Sabat et al. [57], endogenous production of IL-22 initiates the immune system-mediated limitation of adiposity development and metabolic alteration. Further, the authors believe that IL-22 could be used for treatment of the abovementioned disorders.

**Figure 2:**
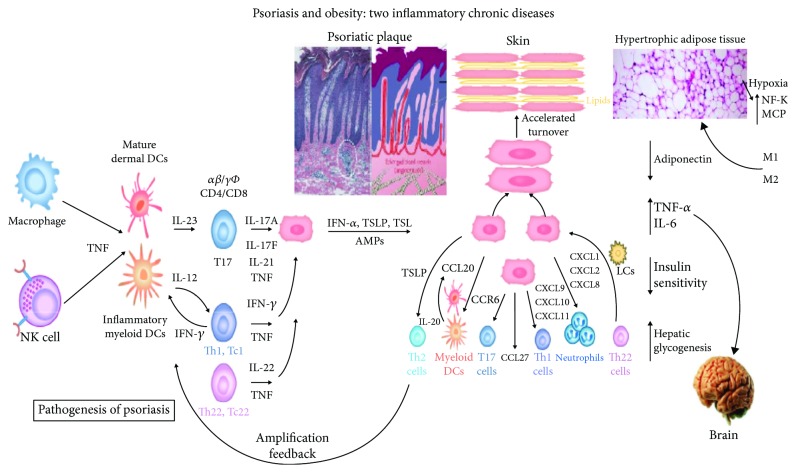
IL-22 stimulation leads to lymphocyte proliferation, which in turn accelerates the synthesis of molecules, elimination of germs, and desquamation. Thus, the epidermis can participate in innate and adaptive immunity in response to infection or stimulation with cytokines. This is due to an increase in the levels of molecules, such as AMP; in the production of chemokines, such as IFN-*α*, TSL, and TSLP; and in the number of Th17 and IL-17-producing CD4+ and CD8+ T cells and T- helper cells (Th). This mechanism responds to the pathogenesis of psoriasis.

Upon activation, keratinocytes synthesize thymic stromal lymphopoietin (TSLP), which is considered an initiator of Th2-mediated immune responses in the skin [58] (Figure 2).

Adipocytes and cells residing within the adipose tissue secrete various soluble mediators involved in regulating organ function, metabolism, immunity, and inflammation. The plasma levels of adiponectin increase with weight loss and decrease in obesity. Adiponectin is an important regulator of metabolism and energy homeostasis, enhancing insulin sensitivity and decreasing hepatic glycogenesis.

In this study from Batycka-Baran et al. [59], it included 30 patients diagnosed as chronic plaque psoriasis and 30 healthy controls. Psoriasin, koebnerisin, human IL-23, and IL-12 were measured, and statistical analysis using SPSS version 16 was performed.

Psoriasin, koebnerisin, IL-12, and IL-23 were significantly increased in all cases, and the risk of psoriasis development was directly related to BMI greater than 30. IL-12 was a good predictor of the psoriasis response to treatment, and IL-23 was decreased in psoriatic arthritis. The risk of developing psoriasis was directly related to increases in BMI greater than 30; thus, obesity will play as another factor along with genetic factors in developing psoriasis [60, 61]. Chronic inflammation and hyperhomocysteinaemia may explain the association with atheroma plaque and metabolic syndrome. The pathophysiology of both psoriasis and obesity showed many shared cytokines that are known to contribute to hypertension, dyslipidaemia, and insulin resistance in the metabolic syndrome [59]. Psoriasin and IL-23 will be important new therapeutic targets for patients with skin psoriasis [62].

In this study from Vachatova et al. [63], the authors selected inflammatory markers. Specifically, they extracted blood samples and determined the following: C-reactive protein (CRP) level was assessed by immunonephelometry on an IMMAGE 800 (Beckman, USA), and the results were expressed in milligrams (mg) per litre of serum.

Adiponectin, leptin, resistin, and lipoprotein-associated phospholipase A2 levels were determined using commercial ELISA kits following the manufacturer's instructions. All of them were considered selective inflammatory markers in psoriatic patients.

Another group of researchers led by Kyriakou et al. [64] collected and analysed 38 papers that discussed the levels of these adipokines in psoriasis (26 of these papers about leptin, 15 about resistin, and 25 about adiponectin). All concluded that leptin and resistin had higher levels in patients with psoriasis, compared to the healthy control population, and that adiponectin levels were lower in psoriasis patients than in healthy patients. However, the heterogeneity of these studies was high, since factors such as sex, BMI, or PASI, among others, can affect these levels.

There is significant evidence that systemic inflammation increases cardiovascular risk and leads to metabolic dysregulation in psoriasis.

In this study of cases and controls by Baran et al. [65], they evaluated the serum irisin levels in patients with psoriasis and associated them with disease, inflammatory, and metabolic parameters and topical treatment in 37 patients with flare of plaque psoriasis (35–64) and 15 sex-, age-, and BMI-matched healthy controls. Initial blood samples were taken and another one after 2 weeks of topical treatment (5% salicylic acid ointment and 0,3% anthralin). Irisin serum level was measured using ELISA. There was a significant statistical correlation between serum irisin levels and age or disease duration. The median irisin serum levels in psoriatic patients did not differ compared to the controls; however, it was 2.5 times higher. In patients with psoriasis, serum irisin levels did not correlate with PASI score or BMI. Assessing irisin levels depending on the severity of psoriasis in each group of patients, no statistical correlations in comparison to the controls were noted. There were no significant relations between the study and control groups in terms of liver enzyme activity, glucose, or lipid levels. Further, there were no significant differences between the groups depending on BMI as compared to the healthy subjects.

Myśliwiec et al. [66] conducted a study on the possible correlation that exists between sphingolipids, such as ceramides and sphingosine-1-phosphate, with psoriasis and possible psoriatic comorbidities such as inflammatory (arthritis) and metabolic diseases (overweight and obesity). To carry out this study, 85 patients with plaque-type psoriasis and 32 healthy controls were used, and the sphingosine-1-phosphate analyses were carried out using high-performance liquid chromatography (HPLC) and silica thin-layer chromatography for ceramides. According to the results obtained in these tests and the correlation with patients in the study, it was determined that in those who presented only plaque psoriasis, ceramides were observed to diminish. However, in those who presented with psoriatic arthritis, the values were observed to increase. Likewise, when they evaluated the concentration of ceramide FA-C22, it was observed to be much higher in patients who presented psoriasis with obesity.

In agreement with the previous results, it was determined that changes in the concentrations of certain sphingolipid species aided diagnosis of psoriasis and some of its comorbidities.

Hyperuricaemia is usually associated with skin psoriasis or PsA, and variations in genetic factors, diet habits, and living areas might contribute to the wide range in its prevalence, as a manifestation of metabolic disorders [67].

Lai et al. [68] carried out a multicentre, cross-sectional observational study to assess the prevalence of asymptomatic hyperuricaemia in Hong Kong Chinese patients with PsA and to investigate the associated factors for hyperuricaemia among them. A total of 160 eligible participants were recruited, from May 2016 to August 2017. The frequency of hyperuricaemia was determined by calculating the percentage of patients with SUA level ≥ 360 mol/L in females and ≥420 mol/L in males. Briefly, 46.9% of the patients were overweight and 9.4% were obese. The overweight patients were found to have the strongest association with hyperuricaemia in PsA, and the SUA level was found to have a statistically significant positive relationship with BMI. No associations were found between lipid profile, renal function, enthesitis index, dactylitis count, CRP, tender and swollen joint counts, duration of the psoriatic conditions, or SUA levels.

Hong Kong Chinese PsA patients had 30.6% of hyperuricaemia (considered as high prevalence), and overweight PsA patients were associated with hyperuricaemia, independent of psoriasis, arthritis severity, renal function, and disease duration. Further, BMI was significantly associated with SUA level, with a positive linear relationship [68].

Baran et al. [69] reported that serum lipocalin-2 levels were much higher in psoriatic patients than in controls, although they were not significantly related to the inflammation markers BMI or PASI. They concluded by saying that lipocalin-2 may be a good predictor of psoriasis and cardiovascular risk, but not a reliable indicator of inflammation, severity of psoriasis, or antipsoriatic treatment outcome.

Based on their study, Watarai et al. [70] suggested that nestin- and FABP5-expressing keratinocytes might be an important diagnostic marker of psoriasis.

He et al. [71] believe that IL-21 is associated with disease severity. In addition, they advocate that it plays an important role in its pathogenesis.

### 3.2. Biomarkers for Prognosis

Gerdes et al. [72] conducted a comprehensive review of the relationship between adipokines and psoriasis, highlighting that these bioactive products (adipokines) are directly related between psoriasis and its comorbidities (insulin resistance, obesity, diabetes mellitus type 2, and cardiovascular diseases). In the case of adiponectin (Acrp30), it has been found that their levels are low in psoriasis in correlation with obesity, but not in the case of leptin and resistin, which correlate with their exacerbation.

With respect to visfatin, there is a correlation between increased level of this with severity of disease. Regarding the retinol-binding protein (RBP4) and omentin, a direct relationship with psoriasis has not been found; however, they have been associated with metabolic processes such as diabetes mellitus type 2 [73].

Finally, altered serum values of tumour necrosis factor alpha (TNF-*α*) and interleukin- (IL-) 6 have been found as important markers in obese patients with psoriasis diagnosis. According to the above, they highlighted that these adipokines can serve as biomarkers to determine the degree of disease advancement, the level of risk posed by comorbidities, and the success that can be obtained by different treatment options [74, 75].

In this research with cases and controls, El-Boghdady et al. [76] evaluated psoriatic patients from an Egyptian hospital (2015–2016) and matching apparently healthy volunteers. The objective was to establish if psoriasin, nestin, Krt16, and IL-21 were possible biomarkers of psoriasis and to correlate them with Body Mass Index (BMI), leptin, and resistin (biomarkers of obesity). Additionally, the researchers sought to identify the bidirectional relationship between psoriasis and obesity. Blood collections (5 mL) from the participants were processed using the ELISA test, and BMI and PASI (when psoriasis was present) were calculated.

They observed that leptin and resistin were significantly increased in obese psoriatic patients as well as in obese and psoriatic groups. Among those groups, they were significantly elevated in obese psoriatic subjects, and all psoriatic patients demonstrated a significant raise by 20.3% (leptin) and 12% (resistin) when compared with the obese group. They were significantly correlated with PASI, plasma psoriasin, nestin, Krt16, and IL-21. IL-21 (inflammatory biomarker) was significantly increased in obese, psoriatic, and obese psoriatic patients. This interleukin has a key role in keratinocyte proliferation. Psoriasin and nestin were significantly raised in psoriatic and obese psoriatic patients. Psoriasin causes inhibition of differentiation in the epidermis because it reduces Krt1 and Krt10 expression. BMI has significant correlations with Krt16 and IL-21. Krt16 produces alterations in Krt filament organization, cell adhesion, differentiation, and migration, and PASI remained significantly associated with psoriasin. Finally, resistin showed a significant correlation with psoriasin, IL-21, and leptin [76].

This study shows the possibility of using psoriasin, nestin, Krt16, and IL-21 as biochemical markers of psoriasis and highlights the correlation of these with biomarkers of obesity (BMI, leptin, and resistin). Furthermore, it reveals the bidirectional association between psoriasis and obesity.

Enany et al. [77] conducted a study evaluating the carotid intima media (IMT) and leptin in patients with psoriasis, who are known to have atherosclerosis risk. The study included 50 patients diagnosed with psoriasis and 10 healthy patients as controls. The psoriatic patients presented significantly higher levels of leptin and carotid intima than controls did. To determine this, each patient was evaluated for the following parameters: duration of disease, body mass index, severity index of the area of psoriasis (PASI), systolic blood pressure, diastolic blood pressure, leptin levels, LDL (low-density lipoprotein) cholesterol levels, and triglyceride levels. Further, significant or highly significant correlations between each of them were determined, with the exception of HDL (high-density lipoprotein) cholesterol, clarifying that psoriasis is an independent risk factor for subclinical atherosclerosis and that cardiovascular deterioration is mainly influenced by the severity of disease, serum triglyceride levels, and leptin. The authors proposed that serum levels of leptin and the level of thickness of the carotid middle intima should be routinely evaluated, with the latter involving evaluating hyperlipidaemia, thus reducing the risk of morbidity and cardiovascular mortality and improving the prognosis of psoriasis.

Myśliwiec et al. [78] carried out a study with cases and controls (85 patients with active plaque psoriasis and 32 sex- and aged-matched healthy controls) to evaluate serum concentrations of fatty acids (FAs) and investigate their association with psoriasis activity, markers of inflammation, and possible involvement in psoriatic comorbidity (obesity, type 2 diabetes, and hypertension). The authors found that the total FA concentration was similar in the psoriatic/control group; in particular, FA was different. There was no correlation between total FA concentration and PASI.

Moreover, in the group of psoriatic patients, they observed a significant negative correlation of eicosapentaenoic acid (EPA) and docosahexaenoic acid (DHA) with PASI. There was a positive correlation between PASI and the n-6/n-3 ratio.

Nevertheless, in nonobese psoriatic patients, they observed a negative correlation between DHA, n-3 polyunsaturated FA (n-3 PUFA), and PASI and a positive correlation of the percentage of monounsaturated FA (MUFA) and PASI [78].

In obese psoriatic patients, no significant correlations between FA and PASI were found. Further, there were no significant correlations between other metabolic parameters such as cholesterol, triglycerides, and fasting blood glucose and PASI in nonobese and obese psoriatic groups.

Conversely, in the psoriatic patient groups with or without hypertension, hypertensive patients had significantly higher concentrations of total FA, percentage of SFA, n-3 PUFA, and SFA/UFA ratio. They also had a lower percentage of all PUFA and n-6/n-3 ratio in serum. The psoriatic group with type 2 diabetes compared to nondiabetic patients had significantly higher concentrations of total FA and a lower percentage of PUFA. Psoriatic patients with diabetes were older and had higher BMI than psoriatics without diabetes. Similarly, psoriatic patients with hypertension were older and had higher BMI than those without hypertension [78].

Kustán et al. [79], in a study conducted in Hungary in patients with psoriasis, measured the levels of water-phase proteins, such as high-specificity reactive C-protein (hsPCR) and orosomucoid in serum and urine (sepsis; ORM and u-ORM). To perform the study, 87 patients were used with levels of mild, moderate, and severe psoriasis and 41 healthy patients as controls. As a result, the values of hsPCR and u-ORM were significantly higher in patients with severe psoriasis. Further, they found a higher concentration of u-ORM/u-CREAT (creatinine in urine) in patients with psoriatic arthritis in relation to those without joint involvement. Thus, in addition to hsPCR, u-ORM is considered a noninvasive sensitive inflammatory biomarker to determine the severity of disease.

In a revision carried out by Gisondi and Giromoloni [80], they evaluated psoriatic comorbidities and their impact on atherothrombotic risk factors. Similar to comorbidities affecting psoriasis prognosis, they observed that psoriasis patients had increased risk of atherothrombotic diseases independently from the concomitance of traditional cardiovascular risk factors.

Hyperhomocysteinaemia has been reported in psoriasis patients and was directly correlated with psoriasis severity according to the PASI score, whereas it was inversely correlated with plasma folic acid levels. Increased levels of serum C-reactive protein (CRP) have been reported in patients with active psoriasis [81].

Patients with pustular psoriasis have significant elevated hs-CRP compared with that of plaque psoriasis patients. A direct correlation between CRP and psoriasis severity was found. CRP elevation is attributable (at least partially) to psoriasis inflammation.

Platelet activation has been established in psoriasis patients; the hemostatic balance is deranged toward a prothrombotic state in psoriasis patients, which might be mainly sustained by platelet hyperactivity.

Cytokines can also mediate several metabolic effects that, in the short term, result in an appropriate response to injury or infection; however, on a chronic basis, it can prove detrimental by accelerating the development of atherosclerosis and predisposing to thrombosis [82]. All of them are directly involved in poor prognosis of psoriasis.

Psoriasis is associated with obesity, dyslipidaemia, altered levels of HDL cholesterol, and impaired cholesterol efflux capacity.

Oxidized LDL (oxLDL) contributes to the spread of atherosclerosis and is an important target for the treatment of cardiovascular disease (CVD). The oxidized phospholipids (oxPL) of lipoproteins A (LPa) increase the proinflammatory response and favour the progression of CVD. In addition, low levels of oxidized HDL (oxHDL) are also associated with an increased risk of CVD in a young healthy population [82].

Sorokin et al. [83] studied 232 subjects with psoriasis, with an average PASI of 7.9. Almost half had hyperlipidaemia, of which 68% were under treatment. The levels of triglycerides (TG), LPa, ApoB, oxHDL, and oxLPa were higher than in healthy volunteers. The most severe patients (PASI > 10) had a negative correlation between oxLDL and oxLPa levels with a calcified plaque burden (DCB) and positive correlation between oxHDL levels with a noncalcified plaque burden (NCB), while the inverse occurred in patients with a PASI < 10.

The paraoxonase-1 (PON-1) system determines antioxidant properties and contributes to the CVD risk. Subjects with psoriasis have higher paraoxonase activity compared to healthy individuals; however, arylesterase levels are lower. Lactonase activity does not vary between people with psoriasis and the healthy population. In one part of the study, patients with psoriasis of a mean age of 47 years, with low cardiovascular risk, and treatment with anti-TNF and anti-IL7 were included. After 3–5 months of treatment, there was a significant increase in the levels of TG and LPa and a decrease in the levels of oxHDL with respect to the basal levels. After 1 year of follow-up, a reduction in the total coronary plaque burden was observed due to a reduction in NCB with changes in DCB.

In summary, circulating lipid levels modified by oxidation can serve as useful markers of early atherosclerosis in psoriasis. This result could help to redirect the prognosis of psoriasis [83].

### 3.3. Biomarker for Treatment

Abud El-Hamd et al. [84] evaluated serum levels of YKL-40 in patients with psoriasis vulgaris before and after treatment with narrow-band UVB phototherapy (NB-UVB). Briefly, 30 patients with vulgar-type psoriasis were taken into account for this study, which were evaluated with the severity index of the psoriasis area (PASI) and 20 control patients. Blood was drawn from both groups before and after the NB-UVB phototherapy. The blood was allowed to clot for a period of 30 minutes, followed by quantification of YKL-40 serum levels by the enzyme-linked immunosorbent assay (ELISA). The results determined that the serum values of YKL-40 were higher in patients with psoriasis vulgaris than the control patients, and the values of YKL-40 and PASI decreased significantly in patients with psoriasis vulgaris after NB-UVB phototherapy. Accordingly, the authors proposed YKL-40 as a biomarker to determine the inflammatory degree of psoriasis.

Gege et al. [85] investigated about the retinoic acid-related orphan nuclear receptor gamma (ROR*γ*/ROR*γ*t). ROR*γ*t is a nuclear hormone receptor (NHR), which converts CD4+ immune cells into Th17 cells and is involved in cytokine production. GSK2981278 is a powerful inverse agonist of ROR*γ* and appears to hinder Th17 expression, including the topical targeting of molecules of psoriatic lesions [86].

Aquaporin-3 (AQP3) is a protein that acts as a water channel, permitting water transport and glycerol permeability across cell membranes. It is involved in the regulation of differentiation and proliferation of keratinocytes as its expression is reduced in psoriasis. Targeting dry and itchy skin as one of the prime aspects of psoriasis, AQP3 is believed to be very relevant for its treatment [87].

Ritchie et al. [88] found that galectin-3 participates in cell proliferation, differentiation, and apoptosis and has a central role in skin disease (dermatitis and some skin cancers) as its decrease leads to changes in keratinocyte functioning and characterization through the JNK pathway.

Bilgiç et al. [89] studied the tumour necrosis factor- (TNF-) like weak inducer of apoptosis (TWEAK). TWEAK is a protein under the TNF superfamily that participates in cell growth, in apoptosis, and in various immune responses. It is involved in inflammatory and immune disorders, including atopic dermatitis, psoriasis, and multiple sclerosis. A potential target for inflammatory conditions driven by IL-17 could be the obstruction of TWEAK since it plays a crucial role in IL-17 signalling pathways. Clinical trial data of the anti-TWEAK monoclonal antibody in rheumatoid arthritis patients have shown promising results.

Finally, in this study of cases and controls, Gisondi et al. [90] enrolled 40 patients with chronic plaque psoriasis with the absence of systemic antipsoriatic treatment for at least 2 months before inclusion and 40 controls matched for age, sex, and BMI.

The objective was to measure serum adipokines in patients with plaque psoriasis, and serum levels of chemerin, resistin, and CRP were significantly higher in patients with chronic plaque psoriasis compared with controls independent of age, sex, BMI, and metabolic comorbidities. Further, they observed that chemerin levels were higher in patients with psoriatic arthritis than those without psoriatic arthritis. Chemerin was linearly correlated to CRP and resistin, but not with psoriasis severity measured with PASI or body surface area (BSA). After 2 and 12 months of treatment with infliximab, a significant reduction in chemerin, resistin, and CRP levels, as well as the PASI score, was observed. In contrast, visfatin levels significantly increased after 12 months of infliximab.

The main biomarkers of inflammation involved on diagnose, prognosis, and treatment in obesity-psoriatic patients are collated in Table 2 [54–57, 60, 61, 63–66, 68–70, 72, 76–80, 83–85, 90].

## 4. Conclusions

For some time, the epidemiological association of psoriasis, especially in its severe forms, with various diseases with which it shares a common pathogenic substrate has been known, including the involvement of tumour necrosis factor *α* (TNF-*α*) and different target organs (such as arthritis and Crohn's disease) and the increased risk of coronary heart disease and occlusive CVD. The severity of psoriasis measured by PASI, disease progression, and response to new treatments, with the so-called metabolic syndrome, is characterized by abdominal obesity, dyslipidemia aterogénica, hypertension, insulin resistance with or without glucose intolerance, and a proinflammatory and prothrombotic state as a risk factor for cardiovascular disease. Further, there is evidence that, in psoriasis, chronic inflammation has a pathogenic role in the metabolic syndrome and associated comorbidities, and its proper treatment could contribute to reversing it.

It is an obligation of dermatologists to recognize elements of the metabolic syndrome and to propose to psoriatic patients, in addition to optimal treatment of their psoriasis, changes in life habits, and adequate pharmacological treatments to reduce the risk of cardiovascular morbidity and mortality.

Thus, the patient with moderate-severe psoriasis has become the main therapeutic target. However, little has been documented about biomarkers that can objectively discriminate between states of disease activity. A biomarker of activity reflects an underlying inflammatory process, is reproducible, and allows patient monitoring, which is useful in making therapeutic decisions.

In this study, we considered the principal biomarkers that could discriminate subjects with active disease or remission, prognosis, and response to treatment in obese patients with comorbidities derived from overweight and high BMI.

Further research is required to achieve biomarkers that objectively discriminate the phases of reproducibility and can be applied in different clinical scenarios and by physicians with different levels of training.

## Figures and Tables

**Table 1:** Treatment of psoriasis.

	Mechanisms of action	Administration via
*Conventional therapies*		
Methotrexate [36]	Inhibits replication of T and B lymphocytes and suppresses secretion of various cytokines, including IL-1 (interleukin-1), interferon-gamma, and TNF-alpha.	Oral and subcutaneous
Cyclosposrin A [34, 36]	Inhibits T cell activation by inhibiting interleukin-2 (IL-2) and interferon-gamma production through inhibition of calcineurin.	Oral
Acitretin [36]	Is a second-generation monoaromatic retinoid. It acts by modulating proliferation of epidermal keratinocytes, joining the nuclear receptor RAR or RXR.	Oral
Phototherapy [33, 36]	Causes alteration of the antigen-presenting cell population (Langerhans cells) and modifies intra- and intercellular signalling mechanisms, leading to development of Th2 preferentially to Th1 responses. It also causes apoptosis of activated T lymphocytes.	Ultraviolet A and B radiation

*Biological treatments*		
Infliximab [35, 37]	Mouse antibody to TNF-alpha	Intravenous
Etanercept [35, 37]	Competitive inhibitor of tumour necrosis factor-alpha. It binds to TNF-alpha to inactivate it.	Subcutaneous
Adalimumab [38]	Anti-TNF IgG1 antibody of an entirely human nature, produced in genetically modified CHO cells.	Subcutaneous
Ustekinumab [39]	Is a fully human IgG1*κ* monoclonal antibody that binds with high affinity and specificity to the p40 protein subunit of the human cytokines IL-12 and IL-23.	Subcutaneous
Ixekizumab [40]	Is a humanized monoclonal antibody. The substance acts by blocking interleukin-17, reducing inflammation. The antibody has affinity to the homodimer IL-17A and heterodimer IL-17A/F.	Subcutaneous
Secukinumab [41]	Is a recombinant monoclonal antibody, entirely human, selective to interleukin-17A.	Subcutaneous

*Others*		
Apremilast [42]	Is a novel phosphodiesterase 4 inhibitor.	Oral

**Table 2:** Biomarkers of inflammation in obesity-psoriatic patients.

Type	Biomarkers	References
Diagnosis	C-reactive protein (CRP)	[63]
	Adipokines (adiponectin, leptin, resistin)	[63, 64]
	Signal transducer and activator of transcription (STAT)	[54]
	S100 family proteins (S100A7, S100A8, S100A9)	[54]
	Wnt family membrane 5a (Wnt5a) protein	[54]
	p53/TP53 protein	[54]
	Phospholipase C (PLC)	[54]
	Psoriasin (S100A)	[55]
	Interleukin (IL-22)	[56, 57]
	Interleukins (IL-12, IL-23)	[60]
	Koebnerisin	[55]
	Irisin	[65]
	Sphingolipids (ceramide FA-C22, sphingosine-1-phosphate)	[66]
	Hyperuricemia	[67]
	Interleukin (IL-21)	[71]
	Keratin-16 (Krt16)	[76]
	Lipocalin-2	[69]
	Class VI intermediate filament protein (Nestin)	[70]

Prognosis	Hyperhomocysteinaemia	[80]
	C-reactive protein (CRP)	[80, 81]
	Platelet activation	[80, 82]
	Cytokines (TNF-*α*, IFN-*α*)	[80, 82]
	Interleukins (IL-1, IL-6, IL-17)	[80]
	Dyslipidemia (↑TG, ↑VLDL, ↑LDL, ↓HDL-C)	[80]
	Body mass index (BMI) > 25	[80]
	Metabolic syndrome	[80]
	Habitual smoking	[80]
	Medications (methotrexate, cyclosporine, retinoids, biologics)	[80]
	Adipokines (adiponectin, leptin, resistin)	[72, 77]
	↑Carotid intima-media thickness	[77]
	Fatty acids (MUFA, SFA UFA, PUFA, EPA, DHA)	[78]
	Urinary orosomucoid (u-ORM). ↑Ratio u-ORM/u-CREAT	[78]
	Oxidation-modified lipoproteins (oxLDL, oxLPa, oxHDL)	[83]
	↑Paraoxonase-1 (PON-1) activity	[83]

Treatment	Serum YKL-40	[83]
	Retinoic acid-related orphan nuclear receptor gamma (ROR*γ*/ROR*γ*t)	[85, 86]
	Aquaporin 3 (AQP3)	[87]
	Galectin-3	[88]
	TNF-like weak (TWEAK)	[89]
	C-reactive protein (CRP)	[90]
	Adipokines (chemerin, resistin, visfatin)	[90]
	Psoriasin (S100A)	[60, 61]

## Abbreviations

IFN-*α*: Interferon-*α*
TNF: Tumour necrosis factor
IL: Interleukin
TSLP: Thymic stromal lymphoprotein
IFN-*α*: Interferon-*α*
LCN: Lipocalin-2
LCs: Langerhans cells
CCR: Chemokine receptors
CXCL: C-X-C motif chemokine ligand
CLA: Cutaneous lymphocyte antigen
(CAMP/LL-37): Cathelicidin
CTACK: Cutaneous T cell-attracting chemokine
NF-KB: Nuclear factor kappa B
MCP-1: Monocyte chemoattractant protein 1
CCL: Chemokine (C-C motif) ligand
M: Macrophages (M1>M2).
